# Docking protein domains in contact space

**DOI:** 10.1186/1471-2105-7-310

**Published:** 2006-06-21

**Authors:** Stefano Lise, Alice Walker-Taylor, David T Jones

**Affiliations:** 1 Department of Biochemistry and Molecular Biology, University College London, UK; 2 Department of Computer Science, University College London, UK

## Abstract

**Background:**

Many biological processes involve the physical interaction between protein domains. Understanding these functional associations requires knowledge of the molecular structure. Experimental investigations though present considerable difficulties and there is therefore a need for accurate and reliable computational methods. In this paper we present a novel method that seeks to dock protein domains using a contact map representation. Rather than providing a full three dimensional model of the complex, the method predicts contacting residues across the interface. We use a scoring function that combines structural, physicochemical and evolutionary information, where each potential residue contact is assigned a value according to the scoring function and the hypothesis is that the real configuration of contacts is the one that maximizes the score. The search is performed with a simulated annealing algorithm directly in contact space.

**Results:**

We have tested the method on interacting domain pairs that are part of the same protein (intra-molecular domains). We show that it correctly predicts some contacts and that predicted residues tend to be significantly closer to each other than other pairs of residues in the same domains. Moreover we find that predicted contacts can often discriminate the best model (or the native structure, if present) among a set of optimal solutions generated by a standard docking procedure.

**Conclusion:**

Contact docking appears feasible and able to complement other computational methods for the prediction of protein-protein interactions. With respect to more standard docking algorithms it might be more suitable to handle protein conformational changes and to predict complexes starting from protein models.

## Background

Physical interactions between proteins are central to many cellular processes [1]. For example they are crucial to the functioning of the immune system and are involved in the regulation of enzyme activity. In order to understand how these interactions are related to biological and biochemical processes, structural information about the complex are essential as they reveal the underlying molecular mechanisms [2,3]. Experimental studies, though, are faced with remarkable technical difficulties and the number of solved complexes deposited in the Protein Data Bank (PDB) [4] is still relatively small. Computational methods, if accurate and reliable, could therefore play an important role, both to infer functional properties and to guide new experiments [5,6].

Docking algorithms attempt to predict the native three-dimensional (3D) structure of a complex starting from the atomic coordinates of its constituent proteins, solved in isolation ("unbound") [7]. It is a challenging problem which has attracted a great deal of interest in view also of its potential biomedical applications (e.g rational drug design and protein engineering [8]). A related problem of considerable importance is domain docking where the aim is to predict the structure of a multi-domain protein from the structures of its component domains. As domain interactions often determine protein function (e.g. by creating a binding site), an understanding of how domains combine and assemble is clearly necessary [9-12]. Moreover, with the progress of structural genomics it can be expected that this question will acquire even more relevance. Structural genomics projects are in fact determining a large number of structures, but focusing primarily at the level of individual domains. The structure of most domains will soon be known either directly from experiments or through accurate homology modeling. The challenge will then be to use them to model large, multi-domain proteins [13].

Most docking procedures treat the individual proteins (or protein domains) as rigid bodies and try to orient them so as to optimize their shape and/or chemical complementarity [14]. Surface side-chain rearrangements and possibly some backbone flexibility are introduced only at a final refinement stage. This strategy can be effective in predicting the structure of the complex in cases where proteins undergo limited conformational changes upon binding. It is clearly inadequate in cases with substantial backbone displacement between bound and unbound forms. As highlighted in recent Critical Assessment of PRedicted Interactions (CAPRI) blind trials [15], this is one of the major limitations of present docking algorithms [16].

In general, docking methods greatly benefit from some biological indications on the likely regions or residues involved in the interaction [17]. This information can be used to guide docking calculations, restricting the search of allowed complex configurations or filtering out wrong solutions [18]. Information about interaction sites can sometimes be available from experiments, e.g. site-directed mutagenesis. Alternatively, one can resort to computational methods. These methods are based on structural, physicochemical and evolutionary properties that distinguish binding sites from the rest of the protein surface (e.g. amino acid composition and residue conservation) [19]. Although no single property is able to reliably locate the interface region in an unbound protein, a number of studies have obtained promising results by combining different features [20-23].

It is known that some residues within the binding interface make a dominant contribution to the stability of protein complexes [24]. These residues can be identified experimentally by alanine scanning mutagenesis and have been named "hot spots". When hot spots are mutated a significant drop in binding affinity is observed whereas the effect is negligible for other residues [25]. Their importance is also confirmed by an evolutionary analysis which shows that hot spots tend to be more conserved [26,27]. It has been observed that hot spots are preferentially located either on protrusions ("knobs") or in depressions ("holes") of the protein surfaces and they are coupled across the interface in tight fitting regions that exclude solvent molecules [28,29]. Interestingly, hot spot residues appear to undergo little conformational changes upon binding [29], a property that might facilitate their identification in the unbound state [30,31].

The picture that emerges from previous experimental and theoretical studies is that protein-protein interfaces are highly heterogeneous: they have many packing defects and are locally optimized at just few critical positions [32]. Statistical analysis of entire interfaces might therefore be unable to capture significant differences between binding sites and the rest of the surface [19]. Analysis focused on the residues important for binding might instead be more discriminating. These considerations suggest an alternative strategy to the docking problem: rather than considering the full three dimensional structure of the complex, it might be more effective to predict just few key contacts across the interface. These could then be used to infer the correct relative orientation of proteins.

In this paper we focus on the (intra-molecular) domain docking problem and present a method to predict contacting residues between domain pairs. The method is based on a pairwise contact function (score) that combines structural, physicochemical and evolutionary information. We use a contact map representation to search for the configuration of contacts that maximizes the score. We show that the approach leads to some contacts correctly predicted and that predicted residues tend to be significantly closer to each other than other pairs of residues in the same domains. Moreover we find that predicted contacts can often be used to discriminate the best model (or the native structure, if present) among a set of 10 optimal solutions generated by a standard docking procedure. In the next paragraph we discuss our results, leaving technical details in the Methods section, after the Conclusions.

## Results and discussion

**Figure 1:**
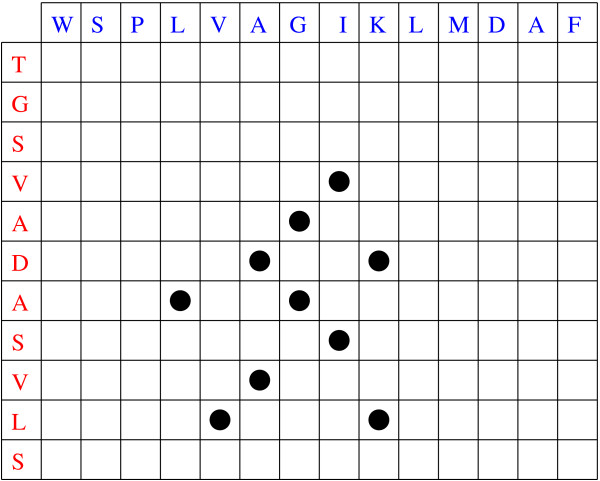
**Contact map**. Schematic representation of a contact map for two interacting domains. Amino acid sequences of the two domains are reported along the axis, in red and blue respectively. Filled circles represent residues in contact.

Contact maps are convenient representations of protein structures that can also be used to describe the interaction between two protein domains (see Fig. 1). In this imaginary example, filled circles correspond to residue pairs in contact across the interface. Within our framework, each contact is assigned a score and the score of a configuration is the sum over all contacts. For simplicity and because we are looking for a few important contacts, we consider maps with a fixed number of contacts, *n*_*c *_= 10. The working hypothesis is that the map configuration that maximizes a suitable scoring function corresponds to the correct interacting residues.

There are a number of advantages in working with contacts maps: it is a simple representation and it should be possible to search the contact space in an efficient manner; a small number of changes on a map may correspond to substantial changes in three dimensions, therefore reducing computational times; in principle any interaction pattern can be represented even allowing for backbone flexibility upon docking. On the other hand, there are some difficulties and limitations: it does not produce directly a 3D complex and more than one structure may correspond to a given contact configuration; if precautions are not taken, many maps do not even correspond to a physically realizable conformation.

**Figure 2:**
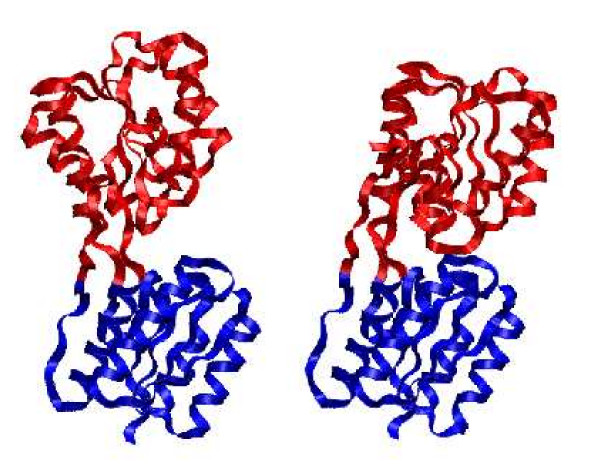
**Open and close conformation**. Ribose-binding protein in its open (left, PDB code 2dri) and close (right, PDB code 1urp) conformation. Domains are identified by different colors.

Docking methods are generally tested on their capacity to predict the protein complex starting from the unbound components [7]. It is simpler but biologically less relevant to reconstruct a complex using the bound structures. The latter are in fact artificially biased toward the native solution. To address this issue, we have selected multi-domain proteins that have been solved experimentally in two conformations which differ for the significant displacement of one of the domains. One conformation can be denoted as "closed", the other as "open" (see Fig. 2). Our data set consists of 20 non redundant domain pairs and the aim is to predict a subset of the contacts in the closed conformation starting from the structure in the open one (in the following we use the PDB code in the closed conformation to identify a protein).

**Figure 3:**
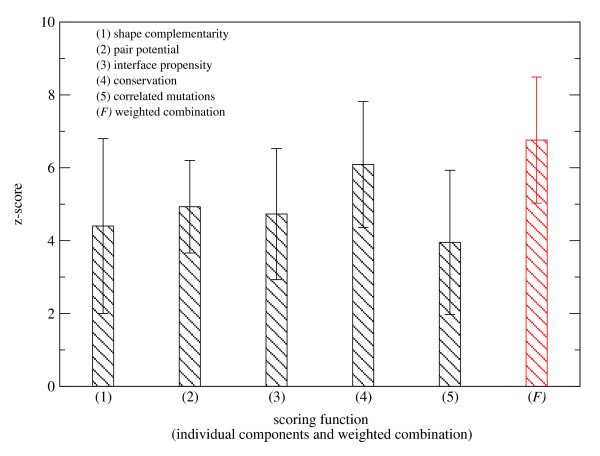
**Average *z*-scores**. Average and standard deviation of *z*-scores for the individual scoring components and for the combined scoring function, calculated over the data set of 20 proteins (*z*-score values corresponding to each single protein are reported in Fig. S1 of Additional File 2).

As detailed in the Methods section, the scoring function is constructed from five different terms: shape complementarity, residue pair potentials, interface propensity, residue conservation and correlated mutations. We have analyzed each scoring component individually and assessed its efficacy in discriminating native contacts from random contacts. In Fig. 3 we report the z-score for each component averaged over the data set, together with the standard deviation (a plot detailing the contributions of each protein can be found in Additional File 2, Figure S1). The *z*-score and standard deviation of the combined scoring function is also plotted.

Residue conservation provides the strongest signal. This is in agreement with a recent study reporting that the number of conserved positions at the interface is significantly higher than on the rest of the protein surface [33]. It should be remarked that statistical analysis performed on the entire interface instead show marginal differences in average conservation between interface and non-interface residues [34]. This apparent discrepancy is likely to derive from the hot-spot organization of interfaces: the few residues important for binding are evolutionary conserved but when averages are taken over the whole interface their distinctive character does not emerge [19,33]. In our approach we consider only the top scoring contacts and this possibly explains the agreement with ref. [33].

The five different components are combined linearly into a unique scoring function. The parameter space of weights has been explored "semi"-exhaustively, i.e. weights are iteratively varied by a factor 2. Given a set of weights, a z-score can be evaluated for each protein in the dataset. We use a leave-one-out cross validation strategy: in turn one protein is singled out (test protein) and the remaining ones are used as the training set. The optimal weights are selected as those that maximize the average z-score on a training set. In Fig. 3 we report the mean and standard deviation of *z*-scores of the combined (optimal) scoring functions, calculated on test proteins. The average value is larger than for individual components although the improvement appears limited e.g. with respect to residue conservation. As a term of comparison we have tested the method with just the residue conservation term in the scoring function.

Weights calculated for different proteins are consistent: the dominant contribution derives from evolutionary information (see Table 4 in the Methods section). Interestingly, correlated mutations have the lowest average *z*-score when considered individually (see Fig. 3) but play an important role in combination with other terms. The weight of residue interface propensity turns out to be negligibly small and accordingly set to zero in our calculations. It is likely that its contribution is already accounted for by the pair potential term. For 15 of the 20 proteins we obtain the same set of parameters: if surface complementarity is given a weight of 1, pair potential, residue conservation and correlated mutations are weighted respectively 2, 8 and 4. These weights are also obtained if the *z*-score average is taken over the whole data set (i.e. no cross validation). Parameters differ in the remaining cases but confirm the importance of evolutionary information.

**Figure 4:**
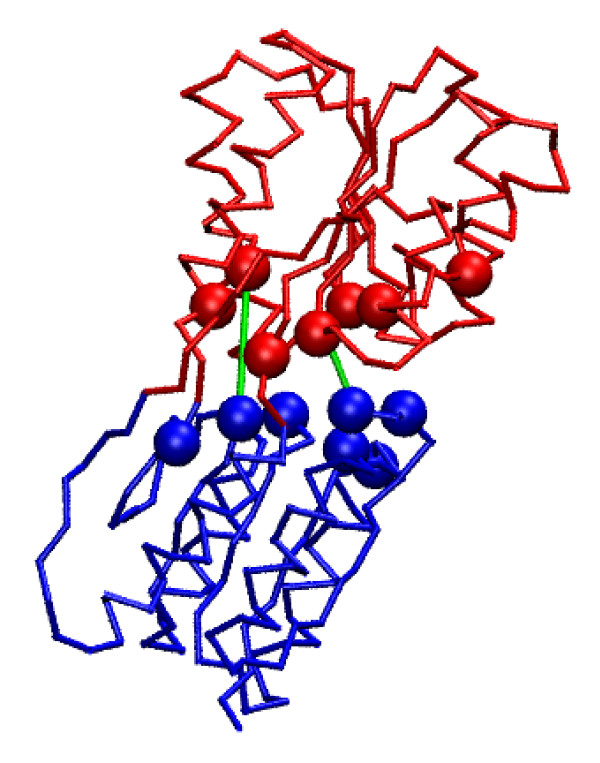
**Contact prediction**. An example of the prediction for ribose-binding protein (PDB code 2dri). Predicted residues are displayed as spheres. Correctly predicted contacts are indicated by a green line joining the residues.

The scoring function is maximized directly in contact space using a simulated annealing algorithm. Some constraints are set on the allowed configurations to avoid unphysical conformations (see Methods for more details). In Fig. 4 we report the result for a specific example (ribose-binding protein, PDB code 2dri). Of the 10 predicted contacts, 2 are correct (i.e. within 5 Å), 4 are within 8 Å and 5 are within 12 Å. The number of correct contacts expected by chance can be estimated from the number of real of contacts, 75, and surface residues (103 and 118 for the two domains respectively). The result is ~6 × 10^-2^. Another quantity of interest is the number of residues that are correctly predicted to be at the interface even if the predicted contact is wrong. In the case of 2dri, 5 out of 7 residues are correctly at the interface in both domains 1 and 2 (note that in general the number of predicted residues at the interface varies because a residue might be be involved in more than one contact).

**Figure 5:**
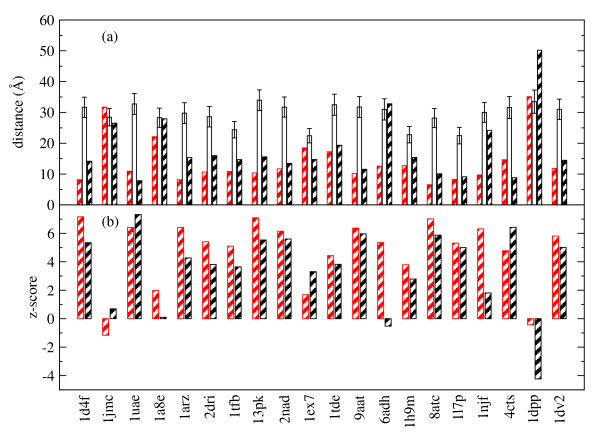
**Average distance of predicted contacts**. In (a) the average distance of predicted contacts in the native structure is reported as obtained for the combined scoring function (red stripe bars) and for the residue conservation term alone (black stripe bars). They are compared to the average and standard deviation of 10 random contacts. In (b) the corresponding *z*-scores are plotted.

The results for each protein in our database are reported in Table 1. The average number of correct predictions is 1.8 which should be compared with an average random expectation of ~5 × 10^-2 ^(the corresponding value for the residue conservation term alone is 1.2). In 14 cases at least one correct contact is identified; in one case (PDB code 8atc) 4 correct contacts are predicted. In general, predicted residues tend to be near the interface and often the binding site is reasonably well located. To illustrate this point we have calculated the average distance of the predicted contacts in the native (closed) configuration, *D*_*pred*_. Fig. 5 reports *D*_*pred *_for each protein (red stripes bars) and compare it with the expected average distance of 10 pairs of residue (one for each domain) picked randomly. If all the predicted contacts were correct then by definition *D*_*pred *_< 5 Å. In our case we obtain that in 15 cases *D*_*pred *_< 15 Å (in 12 cases *D*_*pred*_*<*12 Å) with a significant improvement with respect to random predictions. Fig. 5 also reports the values of *D*_*pred *_for predictions obtained with just the residue conservation term (black stripes bars). It can be noted that in most cases the results are acceptable but worst than those obtained with the combined scoring function.

A second more stringent test is to assess the predicted contacts in relation to the best solutions generated by a standard docking algorithm rather than a set of random contacts. The server GRAMM-X [35] returns 10 possible models of a complex, corresponding to local optima of surface and chemical complementarity. The aim is to verify if the predicted contacts are useful to discriminate the native structure and/or the best available solution in the decoy set (the best model is defined as the one that identifies the largest fraction of native contacts, *f*_*nc*_). To this end, given a prediction of contacts, we have calculated the average distance *D*_*pred *_on each model (besides the native structure) and ranked the solutions in increasing order of *D*_*pred*_. The results for all the proteins in the data set are given in Table 2. The native structure has the lowest *D*_*pred *_in 13 cases out of 20; in 3 cases (PDB codes luae, 9aat and 1h9m) it is ranked second; in 1 case (PDB code 2dri) it is ranked third. For luae and 9aat the structures with lowest *D*_*pred *_correspond to the best models produced by GRAMM-X, which identify a consistent fraction of native contacts (*f*_*nc *_= 0.15 and 0.84 respectively). For 1h9m instead the model ranked first has *f*_*nc *_= 0 but identifies correctly 35% and 40% of interface residues in the two domains; for 2dri the model with lowest *D*_*pred *_has *f*_*nc *_= 0.09 (the best model has *f*_*nc *_= 0.12 and is ranked second).

For 17 targets out of 20 it is possible to define the best GRAMM-X model according to the parameter *f*_*nc *_(in the 3 remaining cases *f*_*nc *_= 0 for all models). For 15 of these targets both the native structure and the best model are ranked within the first three positions in terms of *D*_*pred*_. This suggests that even if the right solution is not present in the decoy set, a solution close to it should be identifiable. In 16 of the 17 cases our contact prediction improves or confirms the GRAMM-X ranking; in the case that is worsened (PDB 1l7p), the best model is re-ranked to second best. We report in Additional File 3 the plot of *D*_*pred *_for each decoy set (Figure S2).

There are cases for which our method does not provide satisfactory results. Two clear examples are PDB codes 1jmc and 1dpp. In the first case, none of the properties we consider or the combined scoring function are able to distinguish clearly between real and random contacts (see Figure S1 in Additional File 2). As a consequence, real contacts do not have particularly high scores and in the search for the maximum are ignored. In the second case, instead, there are correct contacts with relatively high-scores and it is harder to understand the causes of the poor result. One possible explanation is that 1dpp is composed of 3 domains and in our approach we completely ignore one of them, more precisely the intermediate one connecting the N to the C terminal domains. This might introduce some spurious effects as additional interface residues (which in reality are buried) become effectively available for binding. Indeed the predicted contacts appear clustered on the wrong interface. It should be underlined that both 1jmc and 1dpp are likely to be difficult targets. Indeed also GRAMM-X does not perform well: none of the models generated for them has *f*_*nc*_*> *0.

There are other cases where the contact prediction method encounters difficulties, e.g. for 1a8e and 1ex7. It is interesting though that in both cases the predictions, although inaccurate, provide useful indications for selecting an acceptable model among those generated by GRAMM-X. For 1a8e the native structure and the best available model have respectively the lowest and the second lowest value of *D*_*pred*_. For 1ex7, the model ranked second according to *D*_*pred *_has *f*_*nc *_= 0.28 with 75% and 42% of interface residues predicted correctly on the two domains respectively.

## Conclusion

In this work we have presented a novel method to infer contacting residues at domain-domain interfaces. The method attempts to dock protein domains in contact space finding the best configuration of contacts that suits an objective scoring function. It differs and it is complementary to other computational approaches for the prediction of physical protein interactions. In fact it works at an intermediate level between binding site predictions and standard docking algorithms. The former methods attempt to identify the interface residues on a protein without specifying the contacts they actually form, the latter aim to provide a detailed atomic model of the putative complex. Combining and integrating these methods is likely to lead to effective prediction tools. For example, we have shown in this paper that contact predictions can be used in conjunction with the GRAMM-X docking server to discriminate acceptable models. Other methods have also used physicochemical and evolutionary information to improve the ranking of docking solutions [33,36-38]. The emphasis of our work, though, has been more on producing a direct list of putative contacts (and then in case use these to re-rank models). An interesting development would be to guide docking calculations by including from the start the predicted contacts. Provided the predictions are reliable, this would significantly reduce the number of possible complex configurations to be sampled, with clear advantages e.g. in the case of large systems and genome-wide studies. A conceptually similar scheme has been recently proposed [39], in which predicted interface residues (rather than predicted contacts) are used to drive docking calculations.

Overall, contact docking appears feasible and worth considering further. The accuracy of the method is still somewhat limited but amenable to improvements. At present the scoring function is a simple linear combination of five different terms. It is generally recognized that non-linear machine learning algorithms (e.g. neural networks or support vector machine) are more effective in combining heterogeneous sources of information achieving a far higher overall discriminative power. At the same time, some of the individual scoring components might be improved or additional terms included. For example, the description of the energetics of binding is far from adequate as it is based on statistical potentials derived from analysis of entire interfaces and does not include any characterization of binding hot-spots.

We have further shown that contact maps are convenient representations for the docking problem. Contact maps have long being used in the context of single proteins, mainly for structure comparison purposes [40,41]. Some partial success has also been obtained in applying contact maps to predict protein folds [42]. One of the main difficulties in using contact maps for protein folding is to restrict the search to physical maps, i.e. maps that can indeed be reproduced by a 3D protein structure. The same problem recurs in the context of docking as well, i.e. not all maps correspond to a 3D complex, but it is likely to be less severe. In fold prediction, one deals with the protein chain which is quite flexible and in principle can take many different conformations. In docking, although the two domain structures have some degree of plasticity, it is certainly more limited and some geometrical constraints on the allowed contact are easier to introduce. It is clear, though, that the more stringent the constraints the more limited the method will be in handling conformational changes. On the other hand, one can hope that by improving the scoring function, the geometric constraints can be relaxed.

In future work it is our aim to extend the contact docking approach beyond the modeling of multi-domain proteins. We plan to apply the method to the problem of docking two different proteins, though some additional issues will need to be addressed in this case. The correlated mutations analysis, for example, rely on the multiple sequence alignment of co-evolved proteins and on the identification of the correct interacting orthologs. This is a non trivial problem which will require careful consideration. In general, we expect the scoring function will need to be re-adjusted (e.g. the weights). It is also likely that protein conformational changes (upon binding) will be more pronounced. Another direction we plan to explore is the docking of protein models [43]. As the majority of individual protein structures in a genome are going to be models, docking methods will need to be able to handle structural inaccuracies. Contact docking is essentially a low resolution approach and does not depend heavily on structural details. It might therefore be an ideal method for this task. Interestingly, similar considerations could lead to reconsider some of the early docking algorithms [44,45] which, contrary to subsequent developments, were not based on stringent steric match criteria.

## Methods

### Data

Our data set consists of multi-domain proteins that have been solved experimentally in two conformations which differ in the relative orientation of one of the domains. The conformational change brings the domain in closer contact with the rest of the protein. Accordingly, one conformation is denoted as "open", the other as "closed". Proteins have been selected from the Database of Macromolecular Movements [46] and from an analysis of the structural classification database CATH [47]. We have found 20 non redundant examples for which a number a sequence homologs are known. The list is reported in Table 3: 18 are two-domain proteins and 2 are three-domain proteins. In the latter case, only the two domains most affected by the conformational change have been selected. These are domains that are distant in the open configuration and become more strongly interacting in the closed conformation.

The dataset thus comprises 40 domain pairs (20 pairs in the closed conformation and a corresponding set in the open conformation). Ideally, the amino acid sequence of a given domain in the open and closed conformations should be identical. In practice, this is too restrictive and therefore the condition is relaxed to requiring at least 90% sequence identity. Domain definitions and boundaries are taken from CATH which assigns a number to each homologous superfamily. The data set is non-redundant in that no two pairs of interacting domains have the same CATH numbers (at the H-level). Domains from the same superfamily can be present more than once but their domain partners must belong to different superfamilies. Within each superfamily, CATH identifies sequence families (S-level) with a threshold at 35% identity. In our dataset no two domains belong to the same sequence family, i.e. there are no domains sharing more than 35% sequence identity. We report in Additional File 1 the CATH identification numbers (up to the S-level) for the proteins in the dataset.

Interacting domains in the closed configuration must form more than 30 contacts, with each domain having at least 10 residues at the interface (for a definition of inter-domain contacts and interface residues see below). Domains structures generally display some flexibility. In Table 3 we report the *C_α_*-root mean square deviation (RMSD) of the two domains between the open and close configurations. The *C_α_*-RMSD of interface residues is also reported. For these calculations we have used the program ProFit [48]. Domain structures in the open conformations are used as input in our docking calculations. Domains are separated and treated as independent units, disregarding any knowledge of the chain connectivity or on their relative orientation in the open configuration. No prior information on the binding area is assumed. The results are then compared with the protein structures in the close configuration.

Of the 40 protein structures, one has been determined by NMR spectroscopy (PDB code 1tfb). The best model in the ensemble as defined by the NMRCLUST procedure has been selected as the representative [49]. The remaining structures have been solved by X-ray diffraction with a resolution better than or equal to 3.2 Å (the 20 protein structures in the open configuration have been solved with a resolution below 3 Å). For data uniformity, only heavy atoms are considered and no hydrogen atom included. Missing residues and atoms have been modeled with ModLoop [50]. Ligands (cofactors and/or substrates), which are often the cause for the domain motion, are removed for simplicity.

### Surface, interface and contact definitions

Following the convention established at CAPRI [51], two residues in different domains are considered to be in contact if any of their heavy atoms are within 5 Å. Interface residues are defined as those that are involved in at least one contact [52]. Just for the purpose of calculating the interface RMSD, the contact threshold is set to 10 Å and interface residues identified accordingly [51].

The program MSMS is used for molecular surface computations [53]. Given an atomic protein structure, MSMS can produce a triangulated representation of the solvent excluded surface. For each vertex the normal vector to the surface is also calculated. Default values for the radius of the (solvent) probe sphere (1.5 Å) and triangulation density (1.0 vertex/Å^2^) have been used.

Surface atoms are atoms with at least one vertex generated on their van der Waals surface. Residues with one or more surface atoms are surface residues. We have defined a representative point and a representative normal vector for each surface residues. The representative point is given by the geometric average of all vertices generated for that residue (strictly speaking, it might therefore not lie on the protein surface). The representative normal vector is obtained by averaging over all normals associated to that residue.

### Scoring function

The scoring function ℱ
 MathType@MTEF@5@5@+=feaafiart1ev1aaatCvAUfKttLearuWrP9MDH5MBPbIqV92AaeXatLxBI9gBamrtHrhAL1wy0L2yHvtyaeHbnfgDOvwBHrxAJfwnaebbnrfifHhDYfgasaacH8akY=wiFfYdH8Gipec8Eeeu0xXdbba9frFj0=OqFfea0dXdd9vqai=hGuQ8kuc9pgc9s8qqaq=dirpe0xb9q8qiLsFr0=vr0=vr0dc8meaabaqaciaacaGaaeqabaWaaeGaeaaakeaaimaacqWFXeIraaa@3787@ is defined at the amino acid level and assigns a value to each set of 10 contacts. It is a sum of a pairwise contact function *S*_*ij*_,

ℱ=∑n=110Sin,jn
 MathType@MTEF@5@5@+=feaafiart1ev1aaatCvAUfKttLearuWrP9MDH5MBPbIqV92AaeXatLxBI9gBamrtHrhAL1wy0L2yHvtyaeHbnfgDOvwBHrxAJfwnaebbnrfifHhDYfgasaacH8akY=wiFfYdH8Gipec8Eeeu0xXdbba9frFj0=OqFfea0dXdd9vqai=hGuQ8kuc9pgc9s8qqaq=dirpe0xb9q8qiLsFr0=vr0=vr0dc8meaabaqaciaacaGaaeqabaWaaeGaeaaakeaaimaacqWFXeIrcqGH9aqpdaaeWbqaaiabdofatnaaBaaaleaacqWGPbqAdaWgaaadbaGaemOBa4gabeaaliabcYcaSiabdQgaQnaaBaaameaacqWGUbGBaeqaaaWcbeaaaeaacqWGUbGBcqGH9aqpcqaIXaqmaeaacqaIXaqmcqaIWaama0GaeyyeIuoaaaa@482A@

where indices *i*_*n *_and *j*_*n *_refer to residues in the first and second domain respectively. The pairwise contact function *S*_*ij *_is a linear combination of five different contributions (shape complementarity, residue-residue pair potential, residue interface propensity, residue conservation and correlated mutations), which are described below. Since the five components (Sij(k)
 MathType@MTEF@5@5@+=feaafiart1ev1aaatCvAUfKttLearuWrP9MDH5MBPbIqV92AaeXatLxBI9gBaebbnrfifHhDYfgasaacH8akY=wiFfYdH8Gipec8Eeeu0xXdbba9frFj0=OqFfea0dXdd9vqai=hGuQ8kuc9pgc9s8qqaq=dirpe0xb9q8qiLsFr0=vr0=vr0dc8meaabaqaciaacaGaaeqabaqabeGadaaakeaacqWGtbWudaqhaaWcbaGaemyAaKMaemOAaOgabaGaeiikaGIaem4AaSMaeiykaKcaaaaa@33D1@), *k *= 1,..., 5), have different orders of magnitude, they have been rescaled by their standard deviation such that *S*_*ij *_can be written as:

Sij=∑k=15ω(k)(Sij(k)−<S(k)>σ(k))
 MathType@MTEF@5@5@+=feaafiart1ev1aaatCvAUfKttLearuWrP9MDH5MBPbIqV92AaeXatLxBI9gBaebbnrfifHhDYfgasaacH8akY=wiFfYdH8Gipec8Eeeu0xXdbba9frFj0=OqFfea0dXdd9vqai=hGuQ8kuc9pgc9s8qqaq=dirpe0xb9q8qiLsFr0=vr0=vr0dc8meaabaqaciaacaGaaeqabaqabeGadaaakeaacqWGtbWudaWgaaWcbaGaemyAaKMaemOAaOgabeaakiabg2da9maaqahabaacciGae8xYdC3aaWbaaSqabeaacqGGOaakcqWGRbWAcqGGPaqkaaaabaGaem4AaSMaeyypa0JaeGymaedabaGaeGynaudaniabggHiLdGcdaqadaqaamaalaaabaGaem4uam1aa0baaSqaaiabdMgaPjabdQgaQbqaaiabcIcaOiabdUgaRjabcMcaPaaakiabgkHiTiabgYda8iabdofatnaaCaaaleqabaGaeiikaGIaem4AaSMaeiykaKcaaOGaeyOpa4dabaGae83Wdm3aaWbaaSqabeaaieaacqGFOaakieGacqqFRbWAcqGFPaqkaaaaaaGccaGLOaGaayzkaaaaaa@52B3@

where *w*^(*k*) ^are appropriate weights, <*S*^(*k*)^*> *and *σ*^(*k*) ^are respectively the average and standard deviation (over all possible contacts) of the components. In general, several different methods are available for scoring each individual component. Our preference has gone to simple, fairly established methods which have possibly been already tested on docking applications.

#### Shape complementarity

Shape complementarity rewards a contact between protrusions (knobs) and depressions (holes) at domain-domain interfaces. Our approach to locate knobs and holes on domain surfaces is based on a shape function [54] and it is similar in spirit to other methods described in literature [55,56]. Domains are mapped onto a 3D grid, with lattice constant of 0.25 Å. Occupied grid points are defined as those inside the protein domains. They are identified by constructing a set of spheres, one at each protein atom. For surface atoms the sphere radius is the the van der Waals radius of the atom. For interior atoms, the sphere has a radius which is equal to the sum of the atomic van der Waals radius and the probe radius. Grid points that lie inside one of the spheres are considered to be interior points.

To define the shape function, a sphere of radius 6 Å is constructed at each MSMS vertex of the triangulated surface. The shape function at a vertex is then the volume of the sphere that is within the protein domain. The intersection volume is estimated by counting the number of interior grid points. The shape function measures the local convexity of the surface: small values corresponds to knobs, large values to holes. Knobs and holes are identified as vertices at which the shape function is respectively <13
 MathType@MTEF@5@5@+=feaafiart1ev1aaatCvAUfKttLearuWrP9MDH5MBPbIqV92AaeXatLxBI9gBaebbnrfifHhDYfgasaacH8akY=wiFfYdH8Gipec8Eeeu0xXdbba9frFj0=OqFfea0dXdd9vqai=hGuQ8kuc9pgc9s8qqaq=dirpe0xb9q8qiLsFr0=vr0=vr0dc8meaabaqaciaacaGaaeqabaqabeGadaaakeaadaWcaaqaaiabigdaXaqaaiabiodaZaaaaaa@2EA0@*V *and > 23
 MathType@MTEF@5@5@+=feaafiart1ev1aaatCvAUfKttLearuWrP9MDH5MBPbIqV92AaeXatLxBI9gBaebbnrfifHhDYfgasaacH8akY=wiFfYdH8Gipec8Eeeu0xXdbba9frFj0=OqFfea0dXdd9vqai=hGuQ8kuc9pgc9s8qqaq=dirpe0xb9q8qiLsFr0=vr0=vr0dc8meaabaqaciaacaGaaeqabaqabeGadaaakeaadaWcaaqaaiabikdaYaqaaiabiodaZaaaaaa@2EA2@*V*, where *V *is the volume of the 6 Å radius sphere. Moreover, a knob (hole) is selected only if it is a local minimum (maximum). To this end, the shape function at vertices within a distance of 4 Å is checked. A residue is designated as a knob or a hole if one of the vertices on its surface is respectively a knob or a hole. Note that as a consequence of this coarse-grained assignment a residue can carry both labels at the same time. A match between a knob and a hole is rewarded, i.e. Sij(s.c.)
 MathType@MTEF@5@5@+=feaafiart1ev1aaatCvAUfKttLearuWrP9MDH5MBPbIqV92AaeXatLxBI9gBaebbnrfifHhDYfgasaacH8akY=wiFfYdH8Gipec8Eeeu0xXdbba9frFj0=OqFfea0dXdd9vqai=hGuQ8kuc9pgc9s8qqaq=dirpe0xb9q8qiLsFr0=vr0=vr0dc8meaabaqaciaacaGaaeqabaqabeGadaaakeaacqWGtbWudaqhaaWcbaGaemyAaKMaemOAaOgabaGaeiikaGIaem4CamNaeiOla4Iaem4yamMaeiOla4IaeiykaKcaaaaa@36F8@ = 1 if *i *is a knob and *j *a hole (or viceversa), Sij(s.c.)
 MathType@MTEF@5@5@+=feaafiart1ev1aaatCvAUfKttLearuWrP9MDH5MBPbIqV92AaeXatLxBI9gBaebbnrfifHhDYfgasaacH8akY=wiFfYdH8Gipec8Eeeu0xXdbba9frFj0=OqFfea0dXdd9vqai=hGuQ8kuc9pgc9s8qqaq=dirpe0xb9q8qiLsFr0=vr0=vr0dc8meaabaqaciaacaGaaeqabaqabeGadaaakeaacqWGtbWudaqhaaWcbaGaemyAaKMaemOAaOgabaGaeiikaGIaem4CamNaeiOla4Iaem4yamMaeiOla4IaeiykaKcaaaaa@36F8@ = 0 otherwise.

#### Pair potentials

Residue-residue pair potentials are taken from the RPSscore matrix [57]. They are empirical potentials derived from a library of protein-protein interfaces. They have been estimated by comparing the observed to the expected frequencies of residue-residue pairs across the interface and therefore represent the likelihood of two residues type to be in contact (potentials of mean force). The matrix favors certain type of contacts (e.g. Trp-Tyr or I1e-Phe) while disfavoring others (e.g. Lys-Lys or Ser-Ser). The potentials have been derived using a distance cut-off for a contact of 4.5 Å rather than 5.0 Å used in this work.

#### Interface propensity

This term represent the propensity of some amino acid types to be at the interface rather than on the rest of the protein surface. A study by Chakrabarti et al [58] based on known protein complexes has identified two distinct regions at the interface: a core of buried residues and a rim of solvent accessible residues. The rim has similar amino acid composition to the rest of the protein surface whereas the core has distinctive composition. The latter for example has an excess of aromatic residues such as Trp and Tyr and a deficit in charged residues such as Glu and Lys. In our work we have used the core residue propensities. The score of a contact has been defined as the sum of the propensities of the two amino acids involved.

#### Residue conservation

Surface residues that are important for binding are often conserved within a protein family. The evolutionary information can be derived from multiple sequence alignments and quantified by a conservation score. We have used the Variability scale provided in the HSSP database [59]. Variability ranges from zero (perfectly conserved positions in the multiple sequence alignment) to 100 (highly variable positions). For homogeneity with the other scoring terms, we have used the negative value of the Variability (ranging therefore from -100 to 0) so that the higher the score the more conserved is the position. The score assigned to a contact is obtained by adding the (negative) Variability scores of the two positions.

#### Correlated mutations

Correlated mutation analysis identifies sequence positions that tend to evolve in a coordinated manner; The rationale is that if two residues are interacting across the domain interface, changes in one of the two will affect the other so that in turn it will be more likely to mutate to compensate. Correlated mutations are detectable in multiple sequence alignments and we have followed the approach introduced in [60] which has later been extended to domain interactions in [61].

Multiple sequence alignments have been taken from the HSSP database and subsequently filtered. A protein sequence has been retained in the alignment only if: (i) the percentage identity is greater than 30% to the seed protein; (ii) it is alignable over at least 80% of the length of the seed protein; (iii) it is less than 95% identical to any other protein in the alignment. Sequences have been analyzed individually in decreasing order of sequence similarity to the seed protein. They have been added to the filtered alignment only if the above conditions are met. Only proteins with at least 40 homologous sequences in the filtered alignment were included in the data set and considered for the correlated mutations analysis.

We use the McLachlan substitution matrix to quantify amino-acid changes in the multiple alignment [62]. The McLachlan matrix assigns similarity values (≥ 0) between residues. Each column in the alignment is therefore characterized by a set of similarity values, representing all amino-acid pairs observed at that position (a similarity of zero is assigned if a gap is involved). A correlation coefficient between similarity sets at different positions can be calculated. This correlation value ranges from -1 to +1 with a score of +1 indicating highly co-varying positions. As in [63], the correlation value is set to -1 if one of the two positions analyzed has a percentage of gaps > 10%; it is set to 0 if one position is perfectly conserved (and the other has not more than 10% gaps).

#### Weight optimization and cross validation

Given a set of weights, {*w*^(*k*)^}, the *z-score *for each domain pair can be calculated. The score of the 10 best contacts among real contacts, ℱ(max⁡)
 MathType@MTEF@5@5@+=feaafiart1ev1aaatCvAUfKttLearuWrP9MDH5MBPbIqV92AaeXatLxBI9gBamrtHrhAL1wy0L2yHvtyaeHbnfgDOvwBHrxAJfwnaebbnrfifHhDYfgasaacH8akY=wiFfYdH8Gipec8Eeeu0xXdbba9frFj0=OqFfea0dXdd9vqai=hGuQ8kuc9pgc9s8qqaq=dirpe0xb9q8qiLsFr0=vr0=vr0dc8meaabaqaciaacaGaaeqabaWaaeGaeaaakeaaimaacqWFXeIrdaahaaWcbeqaaiabcIcaOiGbc2gaTjabcggaHjabcIha4jabcMcaPaaaaaa@3D8C@, is compared to the expected score of 10 random contacts,

z−score=ℱ(max⁡)−<ℱ>σℱ,
 MathType@MTEF@5@5@+=feaafiart1ev1aaatCvAUfKttLearuWrP9MDH5MBPbIqV92AaeXatLxBI9gBamrtHrhAL1wy0L2yHvtyaeHbnfgDOvwBHrxAJfwnaebbnrfifHhDYfgasaacH8akY=wiFfYdH8Gipec8Eeeu0xXdbba9frFj0=OqFfea0dXdd9vqai=hGuQ8kuc9pgc9s8qqaq=dirpe0xb9q8qiLsFr0=vr0=vr0dc8meaabaqaciaacaGaaeqabaWaaeGaeaaakeaacqWG6bGEcqGHsislcqWGZbWCcqWGJbWycqWGVbWBcqWGYbGCcqWGLbqzcqGH9aqpdaWcaaqaaGWaaiab=ftignaaCaaaleqabaGaeiikaGIagiyBa0MaeiyyaeMaeiiEaGNaeiykaKcaaOGaeyOeI0IaeyipaWJae8xmHyKaeyOpa4dabaacciGae43Wdm3aaSbaaSqaaiab=ftigbqabaaaaOGaeiilaWcaaa@500D@

where <ℱ
 MathType@MTEF@5@5@+=feaafiart1ev1aaatCvAUfKttLearuWrP9MDH5MBPbIqV92AaeXatLxBI9gBamrtHrhAL1wy0L2yHvtyaeHbnfgDOvwBHrxAJfwnaebbnrfifHhDYfgasaacH8akY=wiFfYdH8Gipec8Eeeu0xXdbba9frFj0=OqFfea0dXdd9vqai=hGuQ8kuc9pgc9s8qqaq=dirpe0xb9q8qiLsFr0=vr0=vr0dc8meaabaqaciaacaGaaeqabaWaaeGaeaaakeaaimaacqWFXeIraaa@3787@> is the average random score and σℱ
 MathType@MTEF@5@5@+=feaafiart1ev1aaatCvAUfKttLearuWrP9MDH5MBPbIqV92AaeXatLxBI9gBamrtHrhAL1wy0L2yHvtyaeHbnfgDOvwBHrxAJfwnaebbnrfifHhDYfgasaacH8akY=wiFfYdH8Gipec8Eeeu0xXdbba9frFj0=OqFfea0dXdd9vqai=hGuQ8kuc9pgc9s8qqaq=dirpe0xb9q8qiLsFr0=vr0=vr0dc8meaabaqaciaacaGaaeqabaWaaeGaeaaakeaaiiGacqWFdpWCdaWgaaWcbaacdaGae4xmHyeabeaaaaa@397C@ its standard deviation (both <ℱ
 MathType@MTEF@5@5@+=feaafiart1ev1aaatCvAUfKttLearuWrP9MDH5MBPbIqV92AaeXatLxBI9gBamrtHrhAL1wy0L2yHvtyaeHbnfgDOvwBHrxAJfwnaebbnrfifHhDYfgasaacH8akY=wiFfYdH8Gipec8Eeeu0xXdbba9frFj0=OqFfea0dXdd9vqai=hGuQ8kuc9pgc9s8qqaq=dirpe0xb9q8qiLsFr0=vr0=vr0dc8meaabaqaciaacaGaaeqabaWaaeGaeaaakeaaimaacqWFXeIraaa@3787@> and σℱ
 MathType@MTEF@5@5@+=feaafiart1ev1aaatCvAUfKttLearuWrP9MDH5MBPbIqV92AaeXatLxBI9gBamrtHrhAL1wy0L2yHvtyaeHbnfgDOvwBHrxAJfwnaebbnrfifHhDYfgasaacH8akY=wiFfYdH8Gipec8Eeeu0xXdbba9frFj0=OqFfea0dXdd9vqai=hGuQ8kuc9pgc9s8qqaq=dirpe0xb9q8qiLsFr0=vr0=vr0dc8meaabaqaciaacaGaaeqabaWaaeGaeaaakeaaiiGacqWFdpWCdaWgaaWcbaacdaGae4xmHyeabeaaaaa@397C@ can be calculated from the pairwise contact function, *S*_*ij*_).

Our criterion for weight optimization has been to maximize the average *z*-score over the protein dataset. The weight for the shape complementarity term is set arbitrarily to 1 without loss of generality. The search for the best weights is then carried out combinatorially, sampling the parameter space in the form *w*^(*k*) ^= 2^*n *^with *n *integer number (-9 ≤ *n *≤ 8). In practice, the range of values considered for each term is more limited, e.g. *n *= 1,..., 8 for the residue conservation component, as one can locate the important region through preliminary searches.

A leave-one-out cross validation strategy is used. This implies removing one protein from the data set and calculating the optimized weights based on the reduced data set (having 19 proteins). These are then used in the scoring function to predict inter-domain contacts in the selected protein. In Table 4 we list the 20 sets of optimal weights so obtained. The weight for the interface propensity component turns out to be negligible, i.e. *w*^(3) ^= 1128
 MathType@MTEF@5@5@+=feaafiart1ev1aaatCvAUfKttLearuWrP9MDH5MBPbIqV92AaeXatLxBI9gBaebbnrfifHhDYfgasaacH8akY=wiFfYdH8Gipec8Eeeu0xXdbba9frFj0=OqFfea0dXdd9vqai=hGuQ8kuc9pgc9s8qqaq=dirpe0xb9q8qiLsFr0=vr0=vr0dc8meaabaqaciaacaGaaeqabaqabeGadaaakeaadaWcaaqaaiabigdaXaqaaiabigdaXiabikdaYiabiIda4aaaaaa@308C@ or 1256
 MathType@MTEF@5@5@+=feaafiart1ev1aaatCvAUfKttLearuWrP9MDH5MBPbIqV92AaeXatLxBI9gBaebbnrfifHhDYfgasaacH8akY=wiFfYdH8Gipec8Eeeu0xXdbba9frFj0=OqFfea0dXdd9vqai=hGuQ8kuc9pgc9s8qqaq=dirpe0xb9q8qiLsFr0=vr0=vr0dc8meaabaqaciaacaGaaeqabaqabeGadaaakeaadaWcaaqaaiabigdaXaqaaiabikdaYiabiwda1iabiAda2aaaaaa@3090@ and therefore set to zero and not reported.

### Contact map representation

Contact maps are two dimensional plots that report contacting residues. In the case of inter-domain contacts between two protein domains, having respectively *n *and *m *residues, the contact map is an *n *× *m *matrix. The matrix cell (*i*, *j*) is occupied if residue *i *in the first domain and residue *j *in the second one are in contact, empty otherwise (see Fig. 1). Each matrix cell is assigned a score and the score of a configuration of contacts is given by their sum. We considered configurations with a fixed number of contacts, *n*_c _= 10 and with no more than two contacts per residue. Only surface residues are considered as potentially interacting and included in the contact map.

Distances and orientations of contacting residues on the two domains should be compatible. As discussed above, we assign to each residue a representative point and a normal. We then introduce some geometrical constraints on pairs of contacts, adapted from [55]. Let *p*_1 _and *q*_1 _be two residues on domain 1 in contact respectively with *p*_2 _and *q*_2 _on domain 2. We denote with *d*^(1) ^the Euclidean distance between *p*_1 _and *q*_1_. The angles formed by the line connecting *p*_1 _and *q*_1 _and each of the respective normals are denoted with αp(1)
 MathType@MTEF@5@5@+=feaafiart1ev1aaatCvAUfKttLearuWrP9MDH5MBPbIqV92AaeXatLxBI9gBaebbnrfifHhDYfgasaacH8akY=wiFfYdH8Gipec8Eeeu0xXdbba9frFj0=OqFfea0dXdd9vqai=hGuQ8kuc9pgc9s8qqaq=dirpe0xb9q8qiLsFr0=vr0=vr0dc8meaabaqaciaacaGaaeqabaqabeGadaaakeaaiiGacqWFXoqydaqhaaWcbaGaemiCaahabaGaeiikaGIaeGymaeJaeiykaKcaaaaa@328A@ and αq(1)
 MathType@MTEF@5@5@+=feaafiart1ev1aaatCvAUfKttLearuWrP9MDH5MBPbIqV92AaeXatLxBI9gBaebbnrfifHhDYfgasaacH8akY=wiFfYdH8Gipec8Eeeu0xXdbba9frFj0=OqFfea0dXdd9vqai=hGuQ8kuc9pgc9s8qqaq=dirpe0xb9q8qiLsFr0=vr0=vr0dc8meaabaqaciaacaGaaeqabaqabeGadaaakeaaiiGacqWFXoqydaqhaaWcbaGaemyCaehabaGaeiikaGIaeGymaeJaeiykaKcaaaaa@328C@; the torsion angle between the two normals with *γ*^(1)^. Similar notations with superscript (2) refers to residues *p*_2 _and *q*_2 _on domain 2. Absolute values of differences between quantities are denoted with a Δ, e.g. Δαp=|αp(1)−αp(2)|
 MathType@MTEF@5@5@+=feaafiart1ev1aaatCvAUfKttLearuWrP9MDH5MBPbIqV92AaeXatLxBI9gBaebbnrfifHhDYfgasaacH8akY=wiFfYdH8Gipec8Eeeu0xXdbba9frFj0=OqFfea0dXdd9vqai=hGuQ8kuc9pgc9s8qqaq=dirpe0xb9q8qiLsFr0=vr0=vr0dc8meaabaqaciaacaGaaeqabaqabeGadaaakeaacqqHuoariiGacqWFXoqydaWgaaWcbaGaemiCaahabeaakiabg2da9maaemaabaGae8xSde2aa0baaSqaaiabdchaWbqaaiabcIcaOiabigdaXiabcMcaPaaakiabgkHiTiab=f7aHnaaDaaaleaacqWGWbaCaeaacqGGOaakcqaIYaGmcqGGPaqkaaaakiaawEa7caGLiWoaaaa@4226@.

Two pairs of contacts, (*p*_1_, *p*_2_) and (*q*_1_, *q*_2_), are considered compatible if:

• *d*^(1)^, *d*^(2) ^≤ 20 Å,

• Δ *d *≤ 8 Å,

• Δ*α*_*p*_, Δ*α*_*q *_≤ 1 radian,

• Δ *w *≤ 1 radian,

• Δ*α*_*p*_, + Δ*α*_*q*_*+ *Δ *w *≤ 2 radians.

Compared to ref [55], these thresholds are more permissive, reflecting the fact that we are working at an amino-acid level and therefore at a lower resolution. They are introduced to filter out pair of contacts that are clearly non geometrically compatible.

### Simulated annealing and Monte-Carlo moves

The total number of configurations to be searched in contact space is potentially vast. As we are looking for the maximum of the scoring function, the configurational space can be significantly reduced by considering only high scoring contacts. These are defined as those with a score Si,j≥ <​S​>+σS2
 MathType@MTEF@5@5@+=feaafiart1ev1aaatCvAUfKttLearuWrP9MDH5MBPbIqV92AaeXatLxBI9gBaebbnrfifHhDYfgasaacH8akY=wiFfYdH8Gipec8Eeeu0xXdbba9frFj0=OqFfea0dXdd9vqai=hGuQ8kuc9pgc9s8qqaq=dirpe0xb9q8qiLsFr0=vr0=vr0dc8meaabaqaciaacaGaaeqabaqabeGadaaakeaacqWGtbWudaWgaaWcbaGaemyAaKMaeiilaWIaemOAaOgabeaakiabgwMiZkaaykW7cqGH8aapcaaMb8Uaem4uamLaaGzaVlabg6da+iabgUcaRmaalaaabaacciGae83Wdm3aaSbaaSqaaiabdofatbqabaaakeaacqaIYaGmaaaaaa@405C@, where <*S*> is the average contact score evaluated over all entries in the contact map and σ _S _is the standard deviation. This corresponds roughly to consider the top 32% contacts.

**Figure 6:**
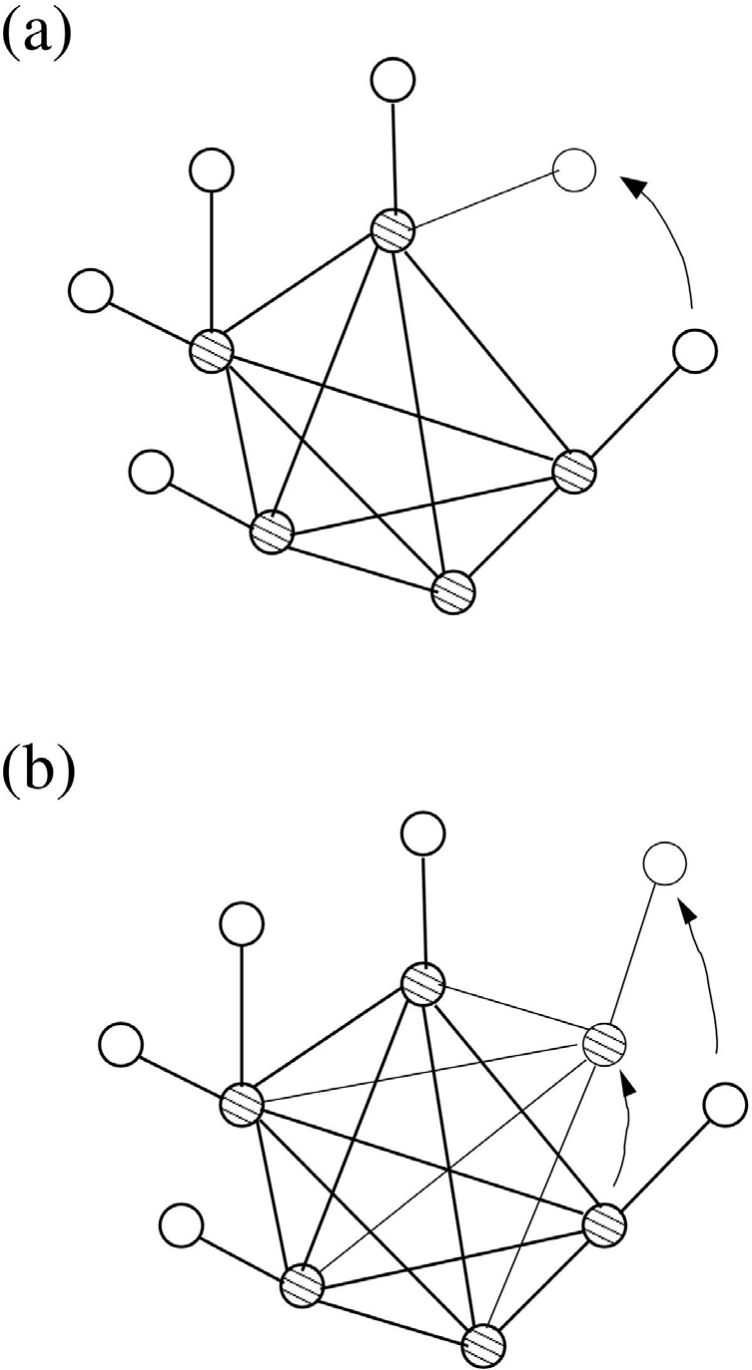
**Monte Carlo moves**. Contact configurations with 5 central nodes (shadowed circles) and 5 peripheral nodes (empty circles). In (a) is displayed a local move, in (b) a large-scale move. Thick lines represent internal connections before the move, thin lines are the new connections after it

The problem can be mapped on a random graph: nodes represent contacts, with an associated weight equal to the contact score; edges between nodes connect pairs of compatible contacts. Typically, 3 – 6% of all possible edges are present in a graph. A clique is a set of nodes with edges between any pair of nodes, i.e. it is a set of mutually compatible contacts. In order to limit the occurrence of non-physical contact configurations we restrict to configurations formed by a central clique of *n *nodes with each of the remaining 10 – *n *nodes connected to at least one of the nodes in the clique. As a convention, in the following we refer to central and peripheral nodes respectively (see Fig. 6). Typically we set *n *= 5 although in some cases we use *n *= 4.

The search for the configuration that maximizes the score is done through a stochastic simulated annealing algorithm. For ease of calculation, we assign each peripheral nodes to one central node (although in principle it might be connected to more than one) and keep track of this relation. We consider two set of Monte Carlo moves, local and large scale, schematized in Fig. 6. Local moves consist of selecting one of the peripheral nodes and replacing it with another that is connected to the central clique (not necessarily connected to the same node). Large scale moves instead select one of the clique nodes and replace it with another node such that the central nodes still form an n-clique. The peripheral nodes attached to the old clique node are also removed and replaced by nodes connected to the new central node. In this manner the structure of a central clique of size *n *with attached peripheral nodes is preserved.

The annealing schedule in the simulation is as follows. Starting with 5-clique dynamics, we first run a cycle of 100*N*_nodes _large-scale moves at infinite temperature (*N*_nodes _is the number of nodes in the graph). We monitor the number of distinct nodes that are visited by the central clique. If the graph coverage is below 70% we turn to 4-clique dynamics and repeat the infinite temperature cycle. This ensures that a consistent fraction of contacts are sampled and that most contact patches are reachable through the clique dynamics. In practice, 4-clique dynamics has been used in 3 cases (PDB codes 1ex7, 1l7p and 1dv2).

The infinite temperature cycle is also used to estimate the largest change in score following a Monte Carlo move, Δℱmax
 MathType@MTEF@5@5@+=feaafiart1ev1aaatCvAUfKttLearuWrP9MDH5MBPbIqV92AaeXatLxBI9gBamrtHrhAL1wy0L2yHvtyaeHbnfgDOvwBHrxAJfwnaebbnrfifHhDYfgasaacH8akY=wiFfYdH8Gipec8Eeeu0xXdbba9frFj0=OqFfea0dXdd9vqai=hGuQ8kuc9pgc9s8qqaq=dirpe0xb9q8qiLsFr0=vr0=vr0dc8meaabaqaciaacaGaaeqabaWaaeGaeaaakeaaimaacqWFXeIrdaWgaaWcbaacbiGae4xBa0Mae4xyaeMae4hEaGhabeaaaaa@3BD6@. The simulation proper is then started at an effective temperature *T *= 10Δℱmax
 MathType@MTEF@5@5@+=feaafiart1ev1aaatCvAUfKttLearuWrP9MDH5MBPbIqV92AaeXatLxBI9gBamrtHrhAL1wy0L2yHvtyaeHbnfgDOvwBHrxAJfwnaebbnrfifHhDYfgasaacH8akY=wiFfYdH8Gipec8Eeeu0xXdbba9frFj0=OqFfea0dXdd9vqai=hGuQ8kuc9pgc9s8qqaq=dirpe0xb9q8qiLsFr0=vr0=vr0dc8meaabaqaciaacaGaaeqabaWaaeGaeaaakeaaimaacqWFXeIrdaWgaaWcbaacbiGae4xBa0Mae4xyaeMae4hEaGhabeaaaaa@3BD6@. Each cycle consist of 100*N*_nodes _large-scale moves with 20(10 – *n*) local moves within any two large-scale moves. Moves are accepted or rejected according to the standard Metropolis test. At the end of a cycle, the temperature is reduced by 10%. The simulation is stopped when no large-scale moves are accepted. A final quenching for the peripheral nodes is then performed. Each simulation is repeated at least 3 times to ensure we obtain consistent results (i.e. we find the same global maximum).

### Result analysis

For each prediction we report *c*_5_, *c*_8_, *c*_12 _which are respectively the number of predicted contacts that are found within 5, 8 and 12 Å in the native structure (*c*_5 _is therefore the number of correct contacts predicted). A quantity of interest is the average distance of predicted contacts in the native structure, i.e.

Dpred=110∑n=110din,jn
 MathType@MTEF@5@5@+=feaafiart1ev1aaatCvAUfKttLearuWrP9MDH5MBPbIqV92AaeXatLxBI9gBaebbnrfifHhDYfgasaacH8akY=wiFfYdH8Gipec8Eeeu0xXdbba9frFj0=OqFfea0dXdd9vqai=hGuQ8kuc9pgc9s8qqaq=dirpe0xb9q8qiLsFr0=vr0=vr0dc8meaabaqaciaacaGaaeqabaqabeGadaaakeaacqWGebardaWgaaWcbaGaemiCaaNaemOCaiNaemyzauMaemizaqgabeaakiabg2da9maalaaabaGaeGymaedabaGaeGymaeJaeGimaadaamaaqahabaGaemizaq2aaSbaaSqaaiabdMgaPnaaBaaameaacqWGUbGBaeqaaSGaeiilaWIaemOAaO2aaSbaaWqaaiabd6gaUbqabaaaleqaaaqaaiabd6gaUjabg2da9iabigdaXaqaaiabigdaXiabicdaWaqdcqGHris5aaaa@4710@

where din,jn
 MathType@MTEF@5@5@+=feaafiart1ev1aaatCvAUfKttLearuWrP9MDH5MBPbIqV92AaeXatLxBI9gBaebbnrfifHhDYfgasaacH8akY=wiFfYdH8Gipec8Eeeu0xXdbba9frFj0=OqFfea0dXdd9vqai=hGuQ8kuc9pgc9s8qqaq=dirpe0xb9q8qiLsFr0=vr0=vr0dc8meaabaqaciaacaGaaeqabaqabeGadaaakeaacqWGKbazdaWgaaWcbaGaemyAaK2aaSbaaWqaaiabd6gaUbqabaWccqGGSaalcqWGQbGAdaWgaaadbaGaemOBa4gabeaaaSqabaaaaa@34FB@ is the distance between residue *i_n _*and *j*_*n*_. Were all the predictions correct then *D*_*pred *_< 5 Å. In general, the smaller *D*_*pred *_the more accurate is the prediction.

Each set of 10 predicted contacts corresponds to two sets of residues, one for each domain. These sets might comprise less than 10 residues because each residue is allowed to have up to two contacts. We denote with *I*_1 _and *I*_2 _the fraction of correctly predicted residues at the interface in the two domains.

The results have been assessed against random predictions of 10 contacts. The expected number of correct contacts obtained by chance can be estimated as c5(r)≃10nn.c.s1s2
 MathType@MTEF@5@5@+=feaafiart1ev1aaatCvAUfKttLearuWrP9MDH5MBPbIqV92AaeXatLxBI9gBaebbnrfifHhDYfgasaacH8akY=wiFfYdH8Gipec8Eeeu0xXdbba9frFj0=OqFfea0dXdd9vqai=hGuQ8kuc9pgc9s8qqaq=dirpe0xb9q8qiLsFr0=vr0=vr0dc8meaabaqaciaacaGaaeqabaqabeGadaaakeaacqWGJbWydaqhaaWcbaGaeGynaudabaGaeiikaGIaemOCaiNaeiykaKcaaOGaeS4qISJaeGymaeJaeGimaaZaaSaaaeaacqWGUbGBdaWgaaWcbaGaemOBa4MaeiOla4Iaem4yamMaeiOla4cabeaaaOqaaiabdohaZnaaBaaaleaacqaIXaqmaeqaaOGaem4Cam3aaSbaaSqaaiabikdaYaqabaaaaaaa@40A1@ where *n*_*n.c *_is the number of native contact and *s*_1 _and *s*_2 _respectively the number of surface residues in domain 1 and 2. The expected average distance and standard deviation of 10 random contact in the native structure can be calculated from the known values of *d*_*i, j*_. The *z*-score of *D*_*pred *_can then be evaluated and its statistical significance assessed. A second test has been carried out with predictions provided by a docking server, GRAMM-X [35]. For each domain pair the server returns a ranked list of 10 possible models of the complex. We have assessed the quality of the models on the basis of the fraction of native residue-residue contacts identified, *f*_*nc*_, which is one the evaluation criteria at CAPRI. High-quality, good and acceptable models have respectively *f*_*nc *_greater than 0.5, 0.3 and 0.1. Other parameters are also used in CAPRI to define the three categories (e.g. backbone and interface root mean square deviations). For simplicity and because *f*_*nc *_is the most pertinent in our context, we have not included them in the discussion.

For 17 of the 20 targets in our data set, GRAMM-X provides at least one model with some native contacts correctly identified; in 7 cases the best model (i.e. the one with highest *f*_*nc*_) is ranked first. For 12 targets the server returns at least one acceptable (*f*_*nc *_> 0.1) solution (5 times the best model is ranked first) and for 5 targets it returns high-quality models (4 times the best model is ranked first). These numbers should not be considered an evaluation of GRAMM-X performances but merely an indication of the non-triviality of our data set.

For each target we have a decoy set composed of the 10 models plus the native structure. Given a contact prediction, the average distance *D*_*pred *_can be evaluated for each model and used to re-rank them. Ideally, the native structure should emerge with the lowest *D*_*pred *_value. Moreover, for the 17 targets which have at least one GRAMM-X model with *f*_*nc *_> 0, the best model should be ranked just after the native structure.

## Authors contributions

SL carried out the study, participated in its design and drafted the manuscript. AWT performed a preliminary investigation during her PhD. DTJ conceived of the study, and participated in its design and coordination and helped to draft the manuscript.

## Supplementary Material

Additional File 1 Table S1 – Data Set. Table containing additional information on the data set used in this study Click here for file

Additional File 2 Figure S1 – *z*-scores. Figure plotting the *z*-scores of the individual scoring components and of the combined scoring function for each protein in the data set. Click here for file

Additional File 3 Figure S2 – *D*_*pred *_values. Plot of the average distance of predicted contacts, *D*_*pred*_, for each decoy set. Click here for file

## Figures and Tables

**Table 1:** Results. Results of contact predictions for the protein in the data set.

PDB code	*c*_5_	*c*_8_	*c*_l2_	*I*_1_	*I*_2_	c5(r) MathType@MTEF@5@5@+=feaafiart1ev1aaatCvAUfKttLearuWrP9MDH5MBPbIqV92AaeXatLxBI9gBaebbnrfifHhDYfgasaacH8akY=wiFfYdH8Gipec8Eeeu0xXdbba9frFj0=OqFfea0dXdd9vqai=hGuQ8kuc9pgc9s8qqaq=dirpe0xb9q8qiLsFr0=vr0=vr0dc8meaabaqaciaacaGaaeqabaqabeGadaaakeaacqWGJbWydaqhaaWcbaGaeGynaudabaGaeiikaGIaemOCaiNaeiykaKcaaaaa@323F@ (× 10^-2^)
1d4f	2	7	9	3/6	6/7	3
1jmc	0	0	0	0/7	1/8	3
luae	3	3	6	7/7	5/9	2
1a8e	0	0	1	3/7	0/7	5
larz	2	6	8	5/7	6/8	3
2dri	2	4	5	5/7	5/7	6
1tfb	1	4	6	5/7	3/6	9
13pk	0	4	7	5/8	6/7	2
2nad	1	4	7	3/5	2/5	2
1ex7	0	0	0	1/6	0/6	6
ltde	1	1	2	2/8	4/6	2
9aat	3	4	5	6/6	5/8	3
6adh	1	3	6	3/6	3/6	3
1h9m	3	4	7	4/6	4/6	12
8atc	4	8	9	6/6	5/8	6
1l7p	1	4	9	5/7	3/5	12
1njf	2	4	8	3/5	2/6	3
4cts	0	3	5	5/7	6/8	4
1dpp	0	0	0	0/7	2/8	3
1dv2	1	2	4	1/5	4/6	2

c5 is the number (out of 10) of predicted contacts that are within a distance of 5 Å (i.e. correctly predicted contacts); c8 and c12 are the numbers of predicted contacts within respectively 8 Å and 12 Å. I1 and I2 are the fractions of correctly predicted residues at the interface in the two domains. 1471-2105-7-310-i1http://www.w3.org/1998/Math/MathMLc5false(rfalse)MathType-MTEF MathType@MTEF@5@5@+=feaafiart1ev1aaatCvAUfKttLearuWrP9MDH5MBPbIqV92AaeXatLxBI9gBaebbnrfifHhDYfgasaacH8akY=wiFfYdH8Gipec8Eeeu0xXdbba9frFj0=OqFfea0dXdd9vqai=hGuQ8kuc9pgc9s8qqaq=dirpe0xb9q8qiLsFr0=vr0=vr0dc8meaabaqaciaacaGaaeqabaqabeGadaaakeaacqWGJbWydaqhaaWcbaGaeGynaudabaGaeiikaGIaemOCaiNaeiykaKcaaaaa@323F@ is the expected number of randomly predicted correct contacts.

**Table 2:** Results for the decoy set.

PDB code	native structure		best model		
	rank	*D_pred_*	rank	*D_pred_*	*f_nc_*

1d4f	1	8.20	2	12.72	0.05
1jmc	9	31.74	-		
1uae	2	10.92	1	10.89	0.15
1a8e	1	22.10	2	23.45	0.17
1arz	1	8.16	2	18.98	0.02
2dri	3	10.67	2	10.35	0.12
1tfb	1	10.81	3	19.37	0.11
13pk	1	10.35	2	11.72	0.05
2nad	1	11.69	3	21.31	0.13
1ex7	6	18.50	2	16.10	0.28
1tde	1	17.24	-		
9aat	2	10.23	1	10.12	0.84
6adh	1	12.57	2	12.69	0.81
1h9m	2	12.72	5	17.27	0.13
8atc	1	6.55	2	8.29	0.82
1l7p	1	8.26	3	8.75	0.82
1njf	1	9.70	2	16.41	0.03
4cts	1	14.64	2	15.17	0.90
1dpp	8	35.12	-		
1dv2	1	11.80	2	15.89	0.08

The decoy set comprises 10 GRAMM-X solutions plus the native structure. The decoys have been ordered in increasing values of *D_pred_*. The rank and the corresponding *D_pred _*of the native structure are reported. Similarly for the best model generated by GRAMM-X, for which *f_nc _*is also shown (*f_nc _*≡ 1 for the native structure). In 3 cases (1jmc, 1tde and 1dpp) is not possible to identify a best model.

**Table 3:** Data set of protein domains.

close conf		open conf		RMSD_1_(Å)	RMSD_2_(Å)	I_RMSD _(Å)
1d4f	A1-A2	1b3r	A1-A2	0.76	0.45	0.67
1jmc	A1-A2	1fgu	A1-A2	1.47	2.18	0.93
1uae	O1-O2	1ejd	A1-A2	0.45	2.34	2.65
1a8e	O1-O2	1bp5	A1-A2	0.54	1.24	1.27
1arz	C1-C2	1dru	O1-O2	1.12	0.35	0.66
2dri	O1-O2	1urp	A1-A2	0.50	0.82	0.81
1tfb	O1-O2	1c9b	A1-A2	3.22	3.21	3.67
13pk	A1-A2	16pk	O1-O2	0.54	1.37	1.71
2nad	A1-A2	2nac	A1-A2	0.41	0.40	0.41
1ex7	A1-A2	1ex6	A1-A2	0.96	0.83	0.94
1tde	O1-O2	1f6m	E1-E2	0.68	1.03	0.71
9aat	A1-A2	lama	O1-O2	0.90	0.33	0.77
6adh	A1-A2	8adh	O1-O2	0.80	0.86	0.91
1h9m	A1-A2	1h9k	A1-A2	0.34	0.60	0.40
8atc	A1-A2	5atl	A1-A2	1.25	2.25	1.09
1l7p	A1-A2	1l7o	A1-A2	0.56	2.82	2.00
1njf	A1-A2	1njg	A1-A2	0.28	0.57	0.63
4cts	A1-A2	1cts	O1-O2	1.14	1.71	1.19
1dpp	A1-A3	1dpe	O1-O3	0.53	0.62	0.78
1dv2	A2-A3	1bnc	A2-A3	0.51	2.55	2.05

PDB codes and domain numbers (according to CATH) of the proteins in the close and open configurations. RMSD_1 _and RMSD_2 _are the C_*α*_-RMSD respectively of domain 1 and 2 between the open and close configurations; I_RMSD _is the C_*α*_-RMSD of interface residues. The last two entries in the table are 3-domain proteins, all others are 2-domain proteins.

**Table 4:** Optimized weights.

PDB code	*w*_2_	*w*_4_	*w*_5_
1d4f	2	8	4
1jmc	2	8	4
1uae	2	8	4
1a8e	2	8	4
1arz	16	32	32
2dri	2	8	4
1tfb	2	8	4
13pk	16	32	32
2nad	2	8	4
1ex7	2	8	4
1tde	1	4	2
9aat	2	8	4
6adh	2	8	4
1h9m	2	8	4
8atc	2	8	4
1l7p	2	8	4
1njf	2	8	4
4cts	16	32	32
1dpp	8	32	16
1dv2	2	8	4

Linear weights of the scoring function obtained with a leave-one-out cross validation. The first column reports the PDB codes; *w*_2_, *w*_4 _and *w*_5 _are respectively the weights associated with pair potential, residue conservation and correlated mutations. The weight of surface complementarity has been set arbitrarily to *w*_1 _= 1; the weight for interface propensity is negligible and therefore ignored (i.e. *w*_3 _= 0).
