# Supplementary material for: Docking protein domains in contact space
Source: BMC Bioinformatics. 2006 Jun 21;7:310. doi: 10.1186/1471-2105-7-310 (PMC1559650; doi:10.1186/1471-2105-7-310)
Supplement: Additional File 1 — Table S1 – Data Set. Table containing additional information on the data set used in this study [file 1471-2105-7-310-S1.pdf]

Table S1 - Data set

The first column list protein PDB codes in the close and open conformation. The number of contacts in the close and open conformations are reported in columns 2 and 3. The number of contacts that are present in the closed conformation but not in the open are in column 4. Columns 5, 6 and 7 report respectively the CATH code (up to the H-level), the number of residues and the number of surface residues in domain 1. Columns 8, 9 and 10 report the analogous information for domain 2. Note that for 1l7p/1l7o only the first 3 CATH numbers have been assigned to domain 2. This does not affect the claim of non-redundancy of the set as no other domain has the same first 3 CATH numbers.

				domain 1			domain 2		
PDB codes	n_{cl}	n_{op}	n_{new}	CATH code	l	s	CATH code	l	s
1d4f/1b3r	73	35	43	3.40.50.1480.1	240	185	3.40.50.720.85	160	130
1jmc/1fgu	34	25	24	2.40.50.140.34	114	103	2.40.50.140.35	124	114
1uae/1ejd	62	28	34	3.65.10.10.3	207	159	3.65.10.10.4	208	163
1a8e/1bp5	75	33	50	3.40.190.10.8	143	123	3.40.190.10.9	165	136
1arz/1dru	44	31	19	3.40.50.720.91	161	136	3.30.360.10.12	98	108
2dri/1urp	75	30	47	3.40.50.2300.10	125	103	3.40.50.2300.11	146	118
1tfb/1c9b	65	24	61	1.10.472.10.11	97	80	1.10.472.10.12	106	86
13pk/16pk	40	20	21	3.40.50.1260.1	188	156	3.40.50.1270.1	208	165
2nad/2nac	54	43	12	3.40.50.720.55	184	148	3.40.50.720.56	190	162
1ex7/1ex6	39	38	15	3.30.63.10.1	60	58	3.40.50.300.46	126	115
1tde/1f6m	34	23	22	3.50.50.60.25	187	159	3.50.50.60.26	128	114
9aat/1ama	69	61	18	3.90.1150.10.6	115	99	3.40.640.10.6	273	223
6adh/8adh	72	63	21	3.90.180.10.1	235	181	3.40.50.720.3	139	116
1h9m/1h9k	55	52	11	2.40.50.100.4	70	67	2.40.50.100.2	71	66
8atc/5at1	104	88	21	3.40.50.1370.3	152	130	3.40.50.1370.4	158	134
1l7p/1l7o	73	70	15	3.40.50.1000.4	145	124	1.10.150.	53	51
1njf/1njg	31	27	10	1.10.8.60.6	73	65	3.40.50.300.84	165	142
1dpp/1dpe	64	39	41	3.90.76.10.2	169	148	3.10.105.10.2	219	170
1dv2/1bnc	32	14	26	3.30.470.20.6	288	243	3.30.1490.20.5	73	70
4cts/1cts	108	102	17	1.10.580.10.2	308	252	1.10.230.10.2	115	102
