# Supplementary material for: Docking protein domains in contact space
Source: BMC Bioinformatics. 2006 Jun 21;7:310. doi: 10.1186/1471-2105-7-310 (PMC1559650; doi:10.1186/1471-2105-7-310)
Supplement: Additional File 2 — Figure S1 – z-scores. Figure plotting the z-scores of the individual scoring components and of the combined scoring function for each protein in the data set. [file 1471-2105-7-310-S2.pdf]

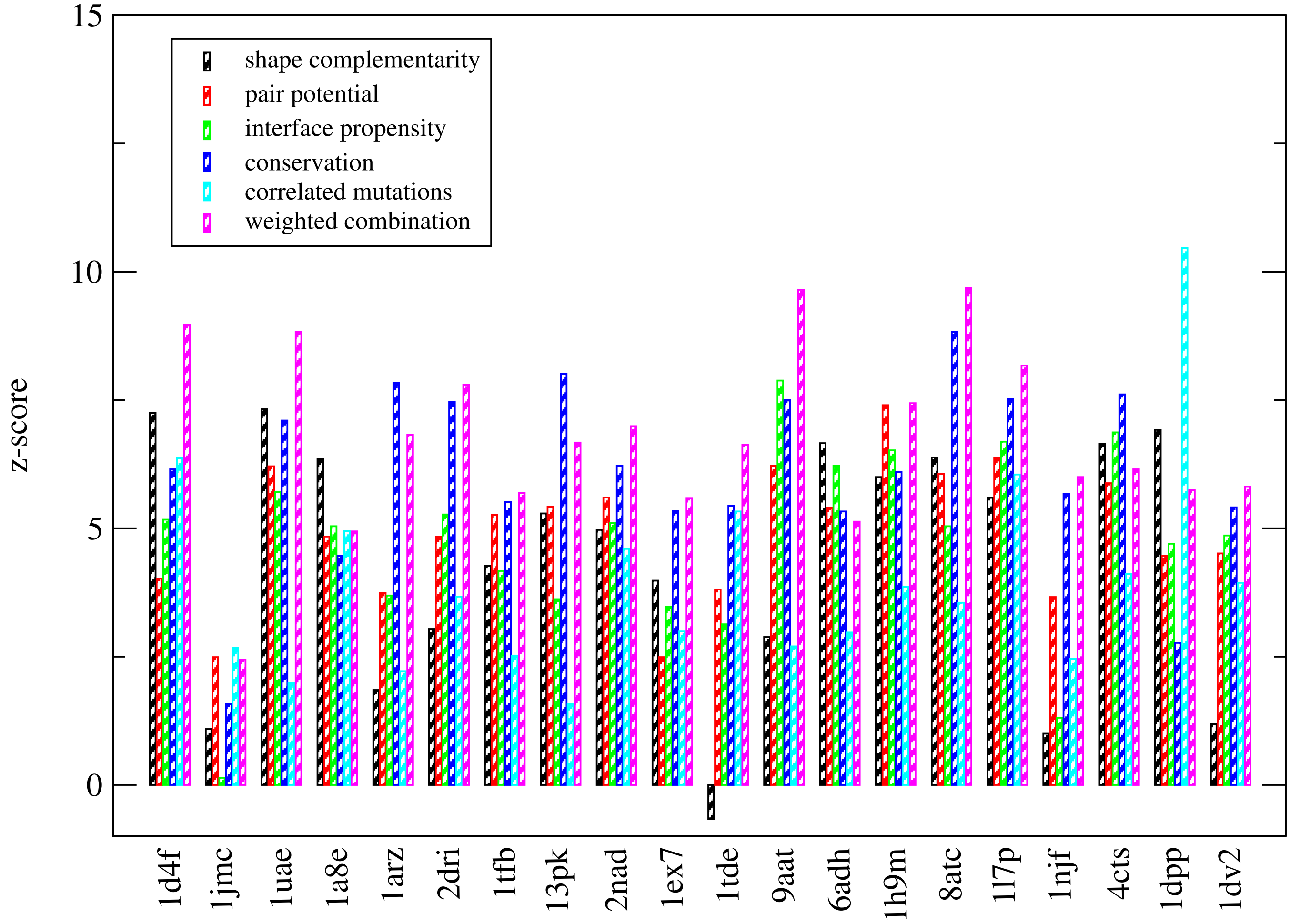

Figure S1 — z-scores

z-scores for the individual scoring components and for the combined scoring function for each protein in the data set.
