# Supplementary material for: Docking protein domains in contact space
Source: BMC Bioinformatics. 2006 Jun 21;7:310. doi: 10.1186/1471-2105-7-310 (PMC1559650; doi:10.1186/1471-2105-7-310)
Supplement: Additional File 3 — Figure S2 – Dpred values. Plot of the average distance of predicted contacts, Dpred, for each decoy set. [file 1471-2105-7-310-S3.pdf]

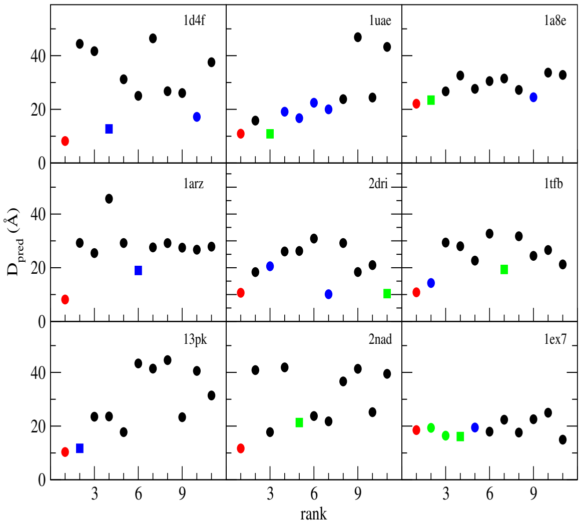

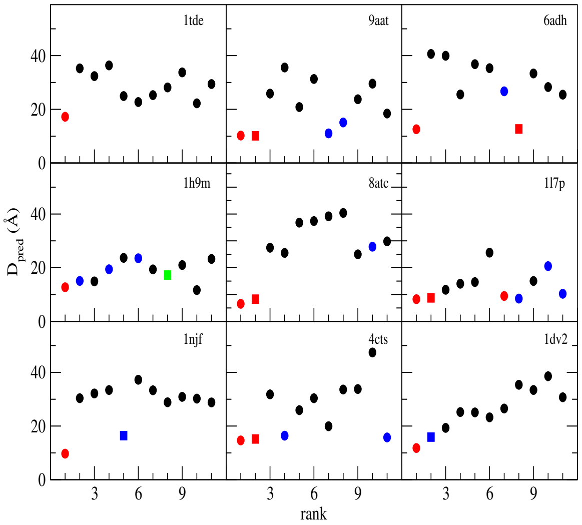

Figure S2 — D_{pred} values

Plot of the average distance of predicted contacts, D_{pred}, for each decoy set. Rank 1 is the native structure; from rank 2 the order is as provided by the server GRAMM-X. Black, blue, green and red symbols correspond respectively to solution with $f_{nc} = 0$, $f_{nc} > 0$, $f_{nc} > 0.1$ and $f_{nc} > 0.5$. The best model provided by GRAMM-X (in terms of f_{nc}) is displayed with a square.
